# Injectable, electrosprayed RGD-coupled alginate hydrogel microcapsules enable enhanced viability and sustained release of mesenchymal stem cells

**DOI:** 10.1007/s10544-026-00815-z

**Published:** 2026-05-07

**Authors:** Dominic Karl M. Bolinas, Allan John R. Barcena, Archana Mishra, Marvin R. Bernardino, Francisco M. Heralde, Steven Y. Huang, Marites P. Melancon

**Affiliations:** 1 Department of Interventional Radiology, The University of Texas MD Anderson Cancer Center, Unit 1471, 1515 Holcombe Blvd, Houston, TX 77030 USA; 2 College of Medicine, University of the Philippines Manila, Manila, 1000 Philippines; 3 The University of Texas MD Anderson Cancer Center UTHealth Houston Graduate School of Biomedical Sciences, Houston, TX 77030 USA

**Keywords:** Mesenchymal stem cells, RGD-Alginate, Electrospray, Hydrogels

## Abstract

The clinical translation of mesenchymal stem cell (MSC) therapies remains limited due to rapid cell clearance and stress-induced viability loss during injection. These limitations emphasize the need to develop delivery systems allowing MSCs to persist in the tissue and exert their biological effect. Cell microencapsulation within alginate (Alg) biomaterials is a promising strategy, where arginine-glycine-aspartic acid (RGD)-coupled alginate (RAlg) hydrogels have recently demonstrated improved bioactivity. However, achieving precise encapsulation and injectability while preserving cell viability remains an ongoing challenge. This study presents an injectable delivery platform using electrosprayed RAlg microcapsules that enhance viability and sustain the release of MSCs. Electrospray parameters were optimized to yield a microcapsule size of 175.4 ± 21.1 μm with high uniformity and consistent spherical morphology. Electron microscopy images of the microcapsules revealed a highly ordered microporous architecture. Physicochemical characterization confirmed that the presence of RGD peptides did not significantly alter the swelling, viscoelasticity, and encapsulation efficiency of Alg. Successful encapsulation of MSCs were observed, with cells assuming a round morphology within the microcapsule. After 14 days, RAlg maintained significantly higher cell viability at 91.3% than Alg alone (84.9%). Furthermore, a time-dependent release of cells was observed, with intact microcapsules at day 1, partial degradation with 59–61% cell release at day 7, and extensive degradation with 78–81% release by day 14. Both RAlg and Alg had comparable release performance. Overall, this study demonstrates the potential of electrosprayed RAlg microcapsules as a biocompatible and injectable platform for the sustained delivery of viable MSCs in regenerative medicine applications.

**Graphical abstract:**

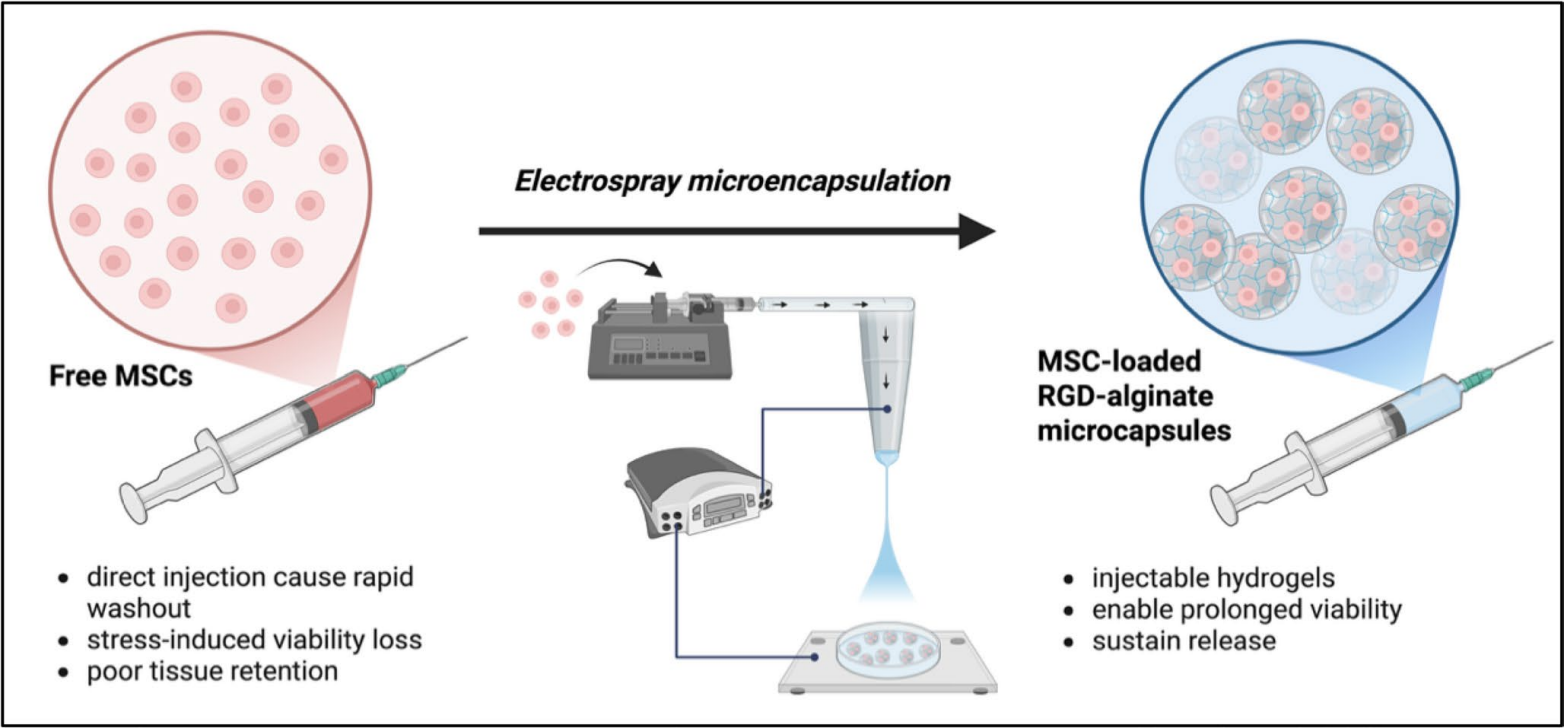

## Introduction

Despite current progress in regenerative medicine, the clinical translation of stem cell therapies remains limited. Mesenchymal stem cells (MSCs) show immense potential for treating chronic conditions such as liver cirrhosis, cardiovascular diseases, and orthopedic injuries (Bagno et al. 2022; Barcena et al. 2024). Although MSCs possess effective regenerative and immunomodulatory properties, they are unable to fully exert therapeutic function clinically even with convincing evidence from preclinical studies (Mousaei Ghasroldasht et al. 2022). A primary cause of this gap is the inefficiency of current administration methods particularly in direct systemic injection (Levy et al. 2020; Tongers et al. 2011). To address these challenges, it is important to identify delivery vehicles that will allow MSCs to survive and stay long enough to promote regeneration and repair.

Conventional delivery techniques provide limited control over cell survival and engraftment at the target tissue. Direct delivery through the systemic circulation often leads to the rapid washout of cells (Lee et al. 2015; Levit et al. 2013). This results to poor cell retention at the target tissue limiting the duration for the MSCs to exert their therapeutic effects (Han et al. 2025). Meanwhile, surgical implantation of preformed constructs increases invasiveness and restricts clinical applicability (Shafiq et al. 2021). Furthermore, viable cell populations decrease due to external mechanical stress and abrupt exposure to hostile local microenvironment (Nevi et al. 2019; Wilson and McDevitt 2013). These limitations emphasize the need for strategies that improve delivery and maximize the functional integration of stem cells.

Cell microencapsulation within biomaterials has been studied as a strategy to develop injectable delivery systems. Biomaterials protect the cells during administration and promote localized retention as compared to direct cell injection (Jha et al. 2015). Among available biomaterials, alginate (Alg) has been widely explored due to their biocompatibility and tunable mechanical properties (Ali and Payne 2021; Mishra et al. 2021, 2023). Under mild gelation conditions, Alg forms hydrogels in versatile morphologies by crosslinking with divalent cations such as calcium. Furthermore, the bioactivity of Alg can be enhanced by incorporation of different polymer blends or cell-adhesion peptides such as arginine-glycine-aspartic acid (RGD) peptides which promote cell survival mechanisms (Barcena et al. 2024; Zdiri et al. 2022). Previous studies have linked the use of RGD-coupled alginate (RAlg) hydrogels scaffolds to enhanced cell viability and growth (Bo et al. 2024; Kudva et al. 2018; Manferdini et al. 2022). However, precise control over the application of RAlg in cell encapsulation while preserving viability remains an ongoing challenge.

Electrospraying is a versatile technique for the robust production of cell-loaded microcapsules with controlled size under gentle processing conditions. By passing a fluid through a high-voltage electric field, electrospraying produces a fine-jet spray of microdroplets that enable efficient encapsulation of living cells while minimizing mechanical stress (Boda et al. 2018; Wang et al. 2019). Optimizing the process parameters, including voltage, flow rate, needle size, and solution concentration, allows rigorous control of the size, uniformity, and sphericity of the microcapsules (Ciarleglio et al. 2024). At optimized parameters, electrospraying maintains the viability, differentiation, immunomodulatory, and pro-reparative capabilities of stem cells (McCrea et al. 2018). When combined with Alg-based hydrogels, electrospraying enables the fabrication of injectable, microscale capsules that provide mechanical protection during delivery and support long-term cell survival of cells (Pangjantuk et al. 2024). Despite these advantages, the integration of electrospraying with injectable Alg systems for stem cell delivery remains underexplored.

Developing an efficient, scalable, and injectable cell delivery system is essential in the clinical translation of current cell-based therapy. We hypothesized that electrosprayed RAlg microcapsules could enable efficient encapsulation, support viability, and sustain release of cells. In this study, we present the first injectable stem cell delivery platform based on electrosprayed RAlg microcapsules and systematically evaluate its performance for cell encapsulation and release. This approach integrates the advantages of electrospraying with the favorable biological properties of RAlg microcapsules, resulting in a minimally invasive, biocompatible system that supports cell survival and controlled release. This study establishes a new, clinically translatable delivery system for enhancing the efficacy of stem cell–based regenerative therapies.

## Materials and methods

### Reagents

Sodium alginate (CAS-No. 9005-38-3), RGD-coupled alginate (CAS-No. 4270-70-1), calcium chloride (CaCl_2_, > 97%, CAS-No. 10043-52-4), phosphate buffered saline (PBS, CAS-No. 24867-26-3) at pH 7.4, sodium citrate (≥ 99.0%, CAS-No. 6132-04-3), trypan blue solution (0.4%, CAS-No. 72-57-1), Dulbecco’s modified eagle medium-high glucose (DMEM), fetal bovine serum (FBS, Gibco, Waltham, MA, USA), and penicillin-streptomycin (10,000 U/mL) were obtained from Sigma-Aldrich (St. Louis, MO, USA).

### Cell culture

The bone marrow-derived Sprague-Dawley red fluorescent protein-MSCs (RFP-MSCs) was purchased from Creative Bioarray (Shirley, NY, USA). A homogenous population of RFP-MSCs from passage 2 was cultured in a complete medium with DMEM, 10% (v/v), FBS, 1% (v/v) penicillin-streptomycin. Cells were incubated at 37˚C in a humidified incubator atmosphere with 5% CO_2_. The media was changed every 3–4 days. All experiments used cells from passage 3–5 at 80–90% confluency.

### Electrospray optimization of microcapsules

Alg microcapsules were produced by electrospraying technology using a standard electrospray system (Spraybase^®^, Cambridge, Massachusetts, USA). The electrospray system consisted of a high-voltage power supply (0–20 ​kV from the emitter to the collector) and a software-controlled, sensitive syringe pump (1.25 × 10^− 5^ mL/min − 1.67 mL/min flow rates using syringes up to 60 mL in size). Electrospraying passed the Alg solution through an electric field, creating a fine spray jet. This process accelerated the formation of smaller and more uniform spray microdroplets. To achieve the optimal microcapsule size of < 200 μm, a combination of the four major parameters was optimized: solution concentration (1.5%, 2%, 2.5%), voltage (6 kV, 7 kV, 8 kV), flow rate (0.05 mL/min, 0.1 mL/min, 0.2 mL/min), and needle size (g22, g24, g26). Prior to electrospraying, the spray needle tip was placed 3 ​cm away from the liquid surface of the gelling solution (100 mM, CaCl_2_) in the collecting dish. Meanwhile, the Alg solution was aspirated in a 3 mL syringe for setup in the syringe pump. Afterwards, the voltage and flow rates were adjusted depending on the tested parameter. The system was in a clean enclosure and allowed to run until all fluid volume was extruded. Through electrospraying, the Alg-based solution was sprayed in a controlled manner directly to the CaCl_2_ solution where crosslinking occurred and producing hydrogels in a microcapsule form. The microcapsules were allowed to crosslink for an additional 10 min, centrifuged at 600 x g for 5 min, and washed with the complete medium through a 70 μm mesh strainer. Recovered microcapsules were stored for further experiments.

### Encapsulation of MSCs

Alg, RAlg, and CaCl_2_ powders were sterilized using ultraviolet (UV) irradiation for one hour. Post-sterilization, a 1.5% solution was prepared by dissolving Alg or RAlg in sterile water and was stirred for one hour at 37 °C. The solution was syringe-filtered using a 0.22 μm filter prior to use. All alginate-based solutions were freshly prepared for every experiment. Next, a confluent passage of RFP-MSCs was harvested and washed three times with sterile PBS and centrifuged at 400 x g for 5 min. The cell pellet was resuspended in the solution to achieve a final cell density of 1 × 10^6^ MSCs. The MSC-loaded Alg/RAlg solution was aspirated in a 3 mL syringe and positioned in the electrospray system with the optimized parameters. The MSC-loaded microcapsules were subsequently washed and used for further experiments.

### Physicochemical characterization of Alg and RAlg

After electrospraying, microcapsules were collected for morphological analysis using the Nikon Eclipse TsR microscope (Nikon, Melville, NY, USA) (Nikon Eclipse TsR, Nikon). A minimum of 100 microcapsules were randomly measured for each group. The mean microcapsule diameter was obtained by measuring the average of two perpendicular diameters crossing the center of all microcapsules using image analysis software (NIS Elements v4, Nikon). Furthermore, the presence of the RFP-MSC within the microcapsule was visualized through fluorescence microscopy using a 560/540 nm excitation filter.

To characterize the ultrastructure of the hydrogel, the microcapsules were imaged using electron microscopy. The microcapsules were fixed using 2.5% glutaraldehyde and underwent a progressive ethanol dehydration process. The processed samples were dried using a critical point dryer and subsequently, sputtered with a 7 nm iridium layer. The microstructure of the gel particles was observed by a scanning electron microscope (SEM) at an accelerating voltage of 5–15 kv under high vacuum. The surface morphology of the microcapsules was visualized through field emission SEM (Nova NanoSEM 230, Fremont, CA, USA). Furthermore, the internal structure and porosity of the microcapsules were visualized through transmission electron microscopy (TEM, JEM 1010, JEOL, Peabody, MA, USA).

For the determination of the viscoelastic properties, Alg solution was loaded and measured using a microviscometer (microVISC, Rheosense, San Ramon, CA, USA). Samples were prepared at varying concentrations and loaded into the viscometer cells (Davarcı et al. 2017). The instrument measured the viscosity range from 0.2 to 20,000 mPa, with an accuracy of 98%. The measurements were performed thrice and the average viscosity was calculated.

The swelling capacity of the microcapsules was assessed using the standard gravimetric method (de Souza et al. 2021). Samples were initially weighed and then incubated at 37 °C in distilled water (50 mL) or media. After 24 h of incubation, the excess liquid was removed, and the samples were re-weighed. The degree of swelling was calculated as follows:

1 $${\%}{S}{w}{e}{l}{l}{i}{n}{g}\:=\:\frac{{Weight}_{24h}-\:{Weight}_{0h}}{{Weight}_{0h}}\:{x}\:100{\%}$$

### Post-encapsulation viability of MSCs

To evaluate the post-encapsulation viability of MSCs, the MSC-loaded microcapsules were placed into 12-well plates and monitored for two weeks. At the designated time points, the microcapsules were incubated with 3.2% sodium citrate for 5 min at 37 °C to dissolve the hydrogels and recover the MSCs. The MSCs was collected, and an equal volume of trypan blue dye was added. The suspension was then loaded into a Cellometer K2 Image Cytometer Automated Cell Counter (Nexcelom Bioscience, Lawrence, MA, USA) for percent (%) cell viability measurement.

### *In vitro* release of MSCs

The release profiles of MSC-loaded microcapsules were evaluated by incubating the microcapsules in phosphate-adjusted culture media (simulating physiological phosphate levels) under serum-free (0% FBS) conditions to deprive the MSCs of mitogenic factors and minimize active proliferation. The MSC-loaded microcapsules were placed on a 50 μm cell strainer and positioned as the upper chamber in a 12-well plate, completed submerged in the media. The released MSCs passed through the upper chamber and settled at the bottom of the receiving well. Concurrently, to quantify the retained cell population, the intact MSC-loaded microcapsules were transferred to a separate plate and subjected to hydrogel dissolution with 3.2% sodium citrate to recover the remaining MSCs. The MSCs were quantified daily to further mitigate possible residual proliferation. The counted cells per day were then aggregated to calculate and report the cumulative cell release twice weekly for 14 days. The percent (%) cell release was calculated as follows:

2 $$\:{\%}{C}{e}{l}{l}\:{r}{e}{l}{e}{a}{s}{e}\:=\:\frac{{N}{u}{m}{b}{e}{r}\:{o}{f}\:{R}{e}{l}{e}{a}{s}{e}{d}\:{C}{e}{l}{l}{s}}{{N}{u}{m}{b}{e}{r}\:{o}{f}\:{R}{e}{l}{e}{a}{s}{e}{d}+{U}{n}{r}{e}{l}{e}{a}{s}{e}{d}\:{C}{e}{l}{l}{s}}\:{x}\:100{\%}$$

### Statistical analysis

All studies were conducted in triplicates (*n* = 3). The data were presented as mean ± standard deviation. GraphPad Prism, version 10.1.2 software (GraphPad, San Diego, CA) was used to perform all statistical analyses. The significance status of variations on the studied responses was evaluated by Analysis of Variance (ANOVA), and a p-value of less than 0.05 was considered to indicate a significant effect.

## Results

### Electrospraying produced tunable, porous, and injectable microcapsules

**Fig. 1:**
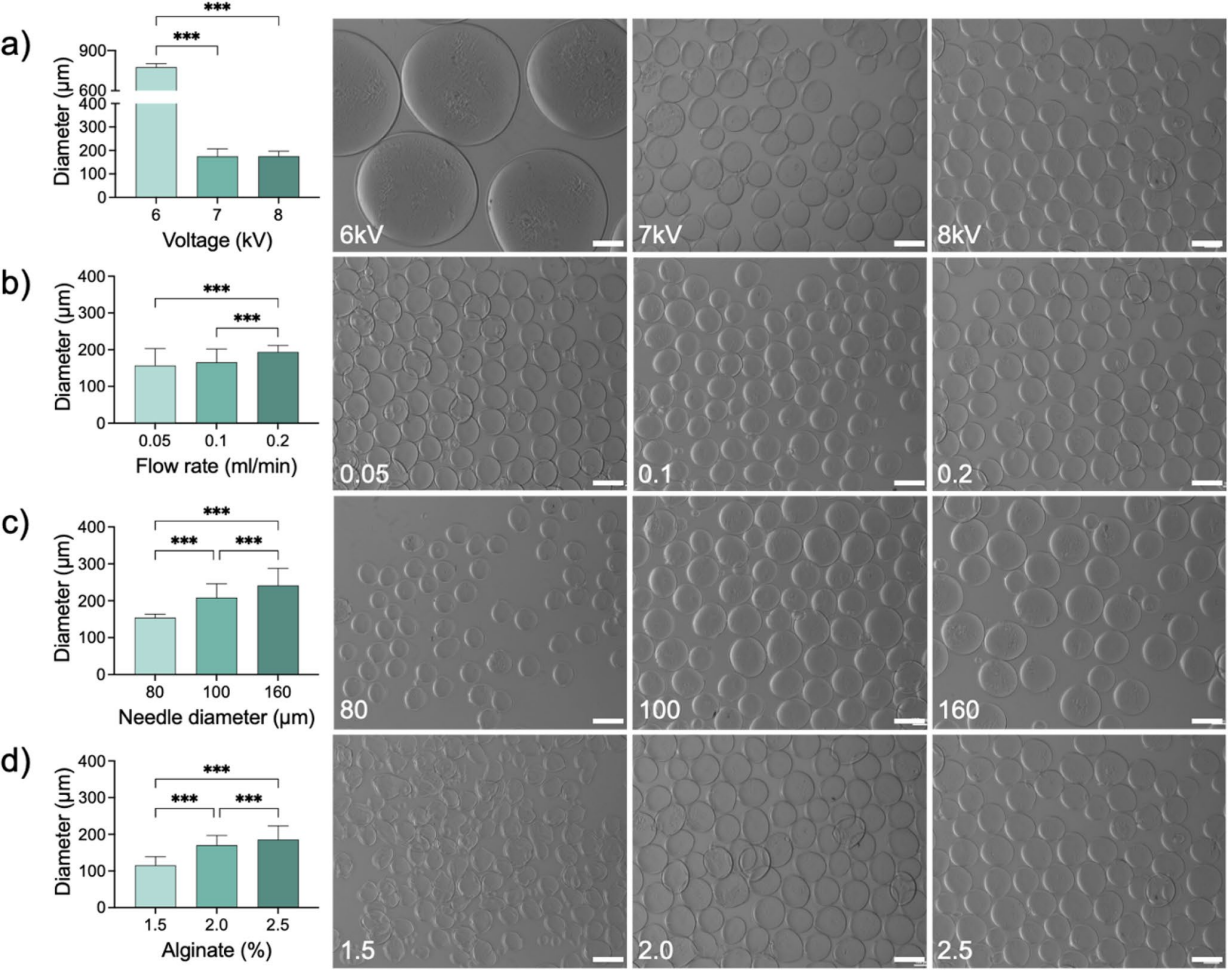
Optimization of Alginate (Alg) microcapsule size using different electrospraying parameters. Systematic variation of applied voltage (**a**), flow rate (**b**), needle diameter (**c**), and Alg concentration (**d**) enabled controlled tuning of microcapsule size as shown in the microscopy images on the right, with higher voltage, lower flow rate, smaller needle diameter, and lower Alg concentration producing smaller microcapsules. Scale bar = 200 μm

To develop injectable hydrogels, electrospraying parameters were optimized to generate microcapsules of the desired size. The target size was set below 200 μm in diameter to balance two key objectives: enabling injection and passage through peripheral capillaries, while providing maximal number of MSCs without causing vascular occlusion. Figure 1 demonstrates the effect of the different electrospraying parameters on microcapsule size. At 7–8 kV, the mean microcapsule size was below 200 μm (174.9 ± 32.1 μm and 175.4 ± 21.1 μm) but at 6 kV the size was 776.0 ± 25.5 μm. Flow rates of 0.05, 0.1, and 0.2 mL/min produced microcapsules with average diameters of 156.7 ± 46.5 μm, 165.7 ± 36.3 μm, and 193.8 ± 17.7 μm, respectively, all below the 200 μm target size. However, slower flow rate led to an increase in size variability as shown by the increase in standard deviation, resulting in greater microcapsule size dispersion. Needle diameter also influenced microcapsule size, with only the 80 μm needle producing microcapsules below 200 μm (154.2 ± 9.8 μm). The effect of the Alg concentration was similarly important where 1.5% (w/v) produced smaller microcapsules (115.3 ± 23.5 μm), but the microcapsules exhibited distorted, non-uniform, nonreproducible, and structurally unstable morphology. In contrast, 2% and 2.5% (w/v) generated spherical microcapsules with diameters of 170.2 ± 26.7 μm and 185.6 ± 37.4 μm, respectively. Based on these results, the optimized parameters selected for subsequent experiments were 7 kV applied voltage, 0.1 mL/min flow rate, 80 μm needle diameter, and 2% Alg concentration, producing sub-200 μm microcapsules with uniform, reproducible spherical morphology.

**Fig. 2:**
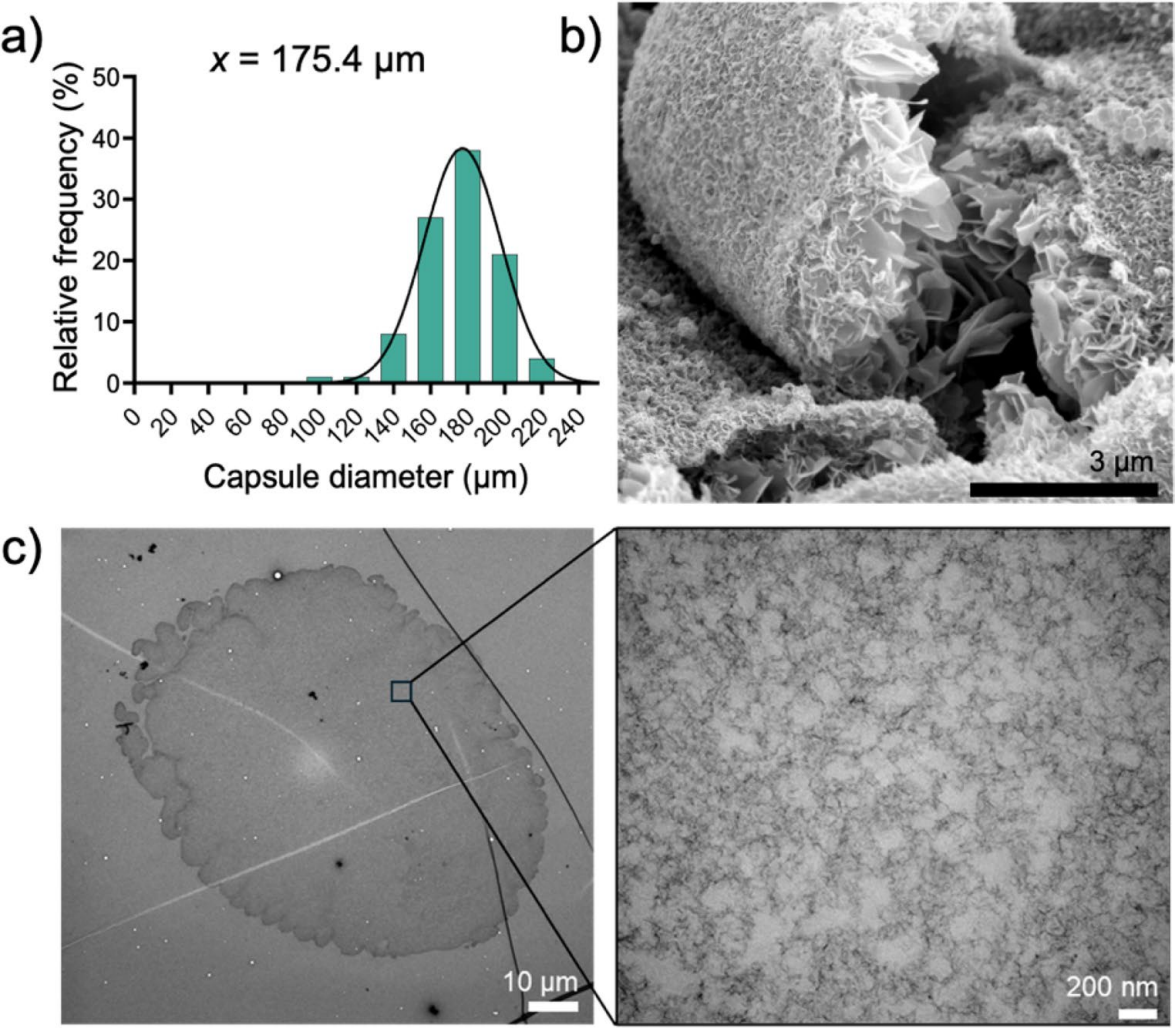
Quantitative and qualitative characterization of alginate microcapsules fabricated under optimized electrospraying conditions. (**a**) Histogram of microcapsule size distribution showing average diameter of 175.4 ± 21.1 μm. Scanning electron microscopy (SEM, **b**) and transmission electron microscopy (TEM) including zoomed-in image (**c**) reveal the surface and internal morphology of the microcapsules, confirming internal porosity conducive to nutrient diffusion

After optimizing the parameters, the obtained Alg microcapsules exhibited a diameter of 175.4 ± 21.1 μm (*n* = 100) with high uniformity as shown Fig. 2a. To further characterize the surface and internal morphology of the microcapsules, SEM and TEM were performed. SEM images showed a rough surface of Alg microcapsules (Fig. 2b). A fractured microcapsule image was captured which revealed plate-like structures that are remnants of the microporous architecture within the microcapsules. This Alg porosity confirmed through TEM where pore width is approximately less than 200 nm (Fig. 2c). Together, these results demonstrate a highly organized hydrogel structure with porosity suitable for cell encapsulation.

**Fig. 3:**
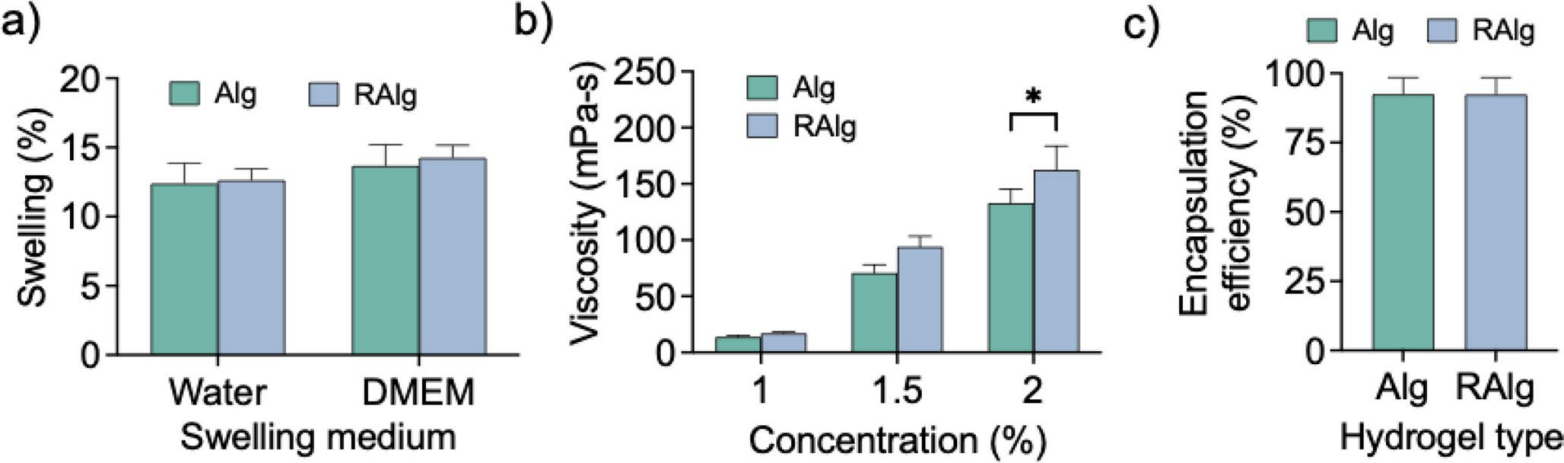
Comparison of the physical characteristics of alginate (Alg) and RGD-coupled Alg (RAlg) microcapsules. (**a**) Swelling behavior was evaluated by submerging microcapsules in water and Dulbecco’s Modified Eagle Medium (DMEM) for 24 h and measuring the weight differences; no significant difference was observed between Alg and RAlg. (**b**) Viscometry analyses showed no significant differences in viscosity between Alg and RAlg at lower concentrations. (**c**) Encapsulation efficiency was assessed by releasing mesenchymal stem cell (MSCs) from the microcapsules and comparing the number of cells released to the initial loading concentration. Both Alg (92.45%) and RAlg (92.31%) microcapsules exhibited high encapsulation efficiency, with no significant difference between groups

RAlg differs from Alg because of the presence of RGD peptides coupled to Alg monomers. To determine whether the presence of RGD peptides impacted the physicochemical properties of Alg, swelling studies, viscometry, and encapsulation efficiency was performed. Swelling studies (Fig. 2a) showed that Alg and RAlg microcapsules exhibited similar fluid uptake over 24 h, with no significant differences in weight increase in either water (12.39% vs. 12.62%, *p* = 0.969) or DMEM (13.67% vs. 14.25%, *p* = 0.827). Viscometry (Fig. 3b) showed similar viscosities for Alg and RAlg with a significant difference emerging at higher concentrations (*p* = 0.021). Encapsulation efficiency (Fig. 3c) was high and similar for both Alg (92.5 ± 6.0%) and RAlg (92.3 ± 6.1%) microcapsules (*p* = 0.9909). In summary, these results show that the presence of RGD peptides in RAlg did not significantly alter the swelling, viscoelasticity (at low concentrations), and encapsulation efficiency of the microcapsules.

### RAlg microcapsules improved cell viability and controlled the release of MSCs

**Fig. 4:**
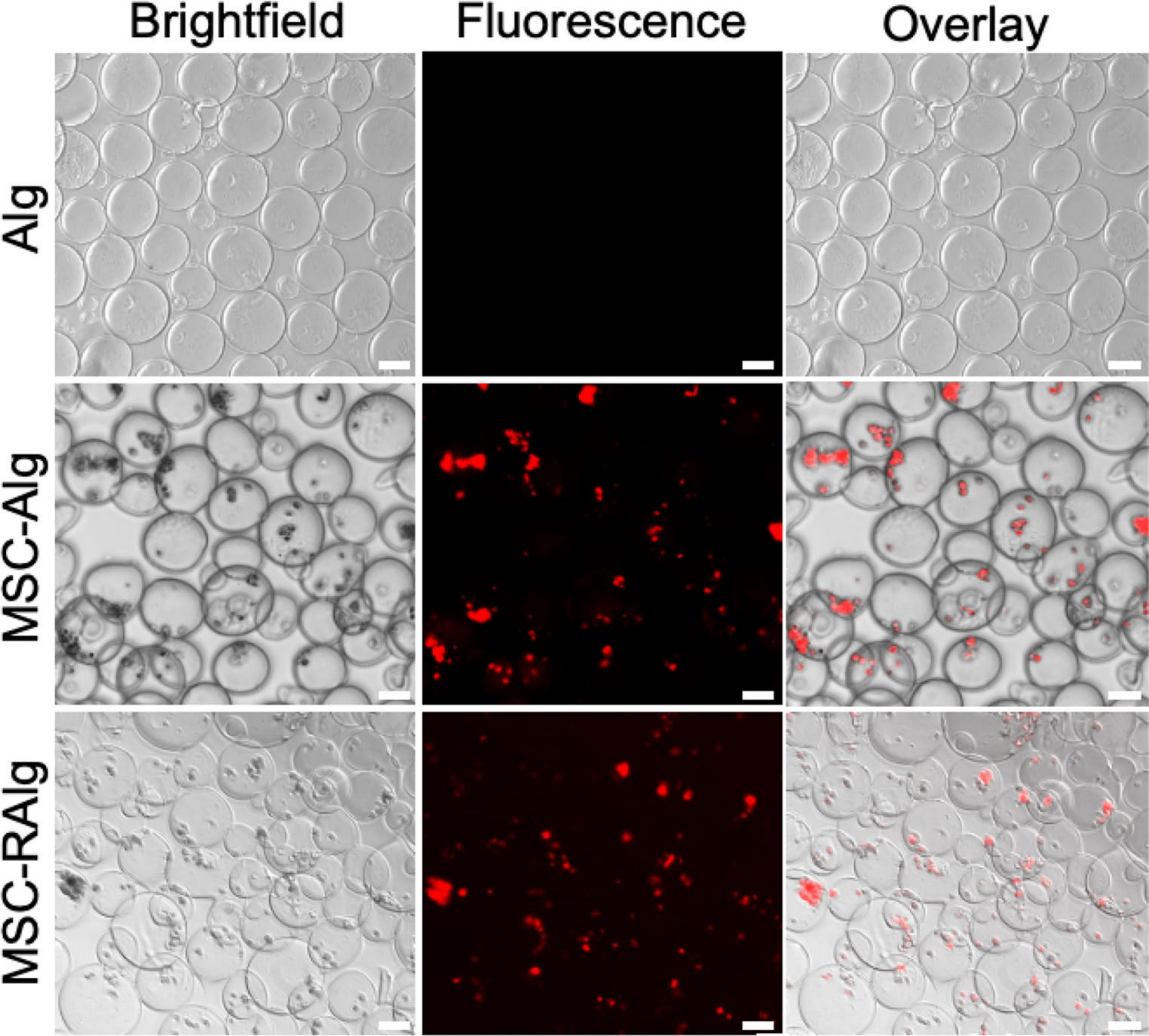
Brightfield and fluorescent microscopy images of MSC-loaded alginate (MSC-Alg) and RGD-coupled alginate (MSC-RAlg) microcapsules. Brightfield microscopy images show microcapsule with spherical shape and an approximate average diameter of 200 μm. Fluorescence microscopy images show red fluorescent protein (RFP)-expressing MSCs encapsulated within Alg-based microcapsules, confirming successful cellular incorporation. Scale bar = 100 μm

**Fig. 5:**
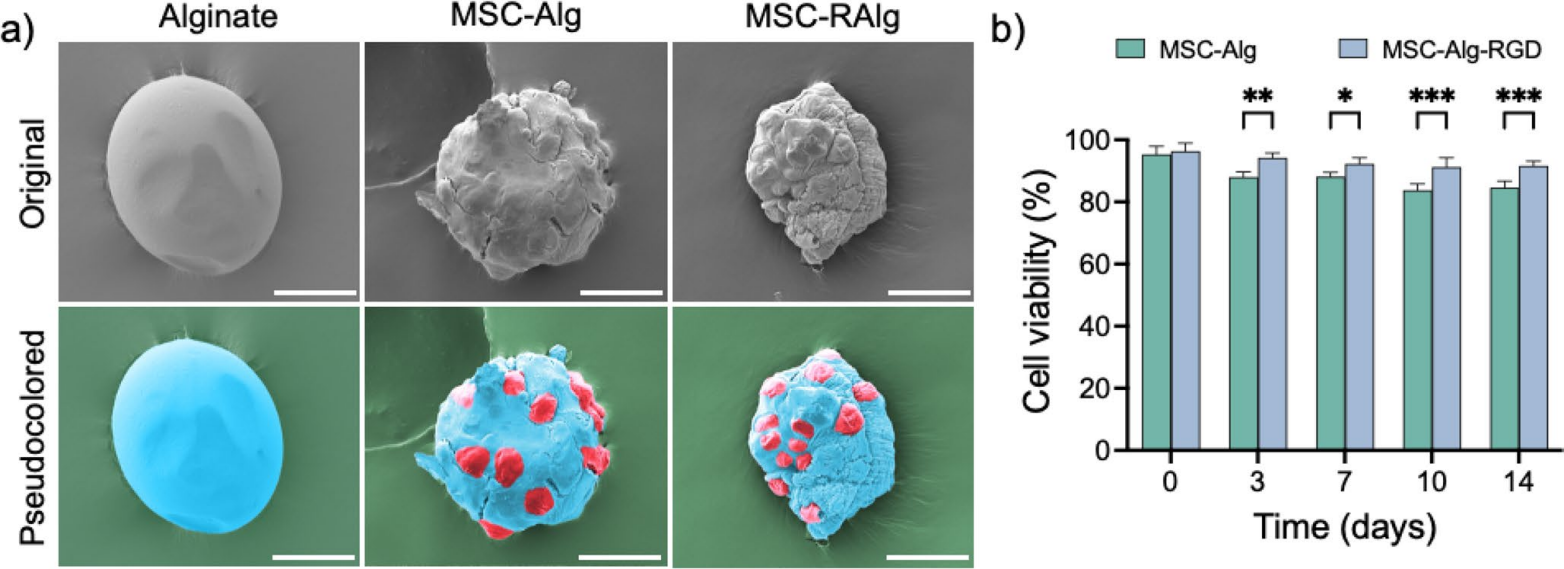
Scanning electron microscopy (SEM) imaging and MSC viability assessment of MSC-loaded alginate (MSC-Alg) and RGD-coupled (MSC-RAlg) microcapsules. (**a**) Representative SEM images of a single Alg and RAlg microcapsules with or without MSCs (pseudocolored red) demonstrated successful loading of Alg-based microcapsules (pseudocolored blue). Surface characterization showed distinct MSC imprints from the interior of the microcapsules. Longitudinal viability of encapsulated MSCs was assessed using trypan blue viability assay over 14 days. (**b**) No significant differences in MSC viability in MSCs were observed between Alg and RAlg microcapsules at early time points. After 14 days, MSC viability in RAlg (91.3%) remained significantly higher than in Alg microcapsules (84.9%). Scale bar = 50 μm

The MSC-loaded microcapsules were visualized using brightfield and fluorescence microscopy (as shown in Fig. 4). After applying a 560/540 nm filter, the RFP expressed in MSC highlighted the presence of cells within the microcapsules. The MSCs assumed a spherical morphology inside the microcapsules in contrast to their original spindle-shape, flat morphology in cell culture. Furthermore, the MSC-loaded microcapsules were processed for electron microscopy to characterize their ultrastructure morphology (Fig. 5a). A thorough optimization of the dehydration process was performed to minimize any significant morphological changes compared to the native state. On imaging, SEM showed globular microcapsules with protruding features outlining the MSCs’ location inside the microcapsules. The original images were pseudocolored to highlight the MSCs (red). The average diameters for the MSC-loaded Alg and RAlg microcapsules are 175 ± 32.1 μm and 170.3 ± 26.7 μm, respectively (*p* = 0.2683). All in all, these findings confirmed that the MSCs were successfully loaded in injectable Alg and RAlg microcapsules.

Following successful MSC encapsulation, the ability of the microcapsules to maintain cell viability was evaluated (Fig. 5b). MSC-loaded microcapsules were cultured for 14 days, and the viability was quantified twice weekly. On each sampling day, microcapsules were dissolved and recovered cells were collected and assessed for viability using trypan blue assay. At baseline, MSC viability exceeded 95% viability in both Alg (95.3%) and RAlg (96.3%). After 3 days, viability of MSCs in Alg decreased to 88.0%, whereas MSCs in RAlg showed minimal reduction to 94.3% (*p* < 0.001). By day 14, MSC viability in Alg further declined to 84.9%, while RAlg microcapsules maintained significantly higher viability at 91.3%. These results indicate that RAlg provides enhanced biological support for MSC viability compared to unmodified Alg.

**Fig. 6:**
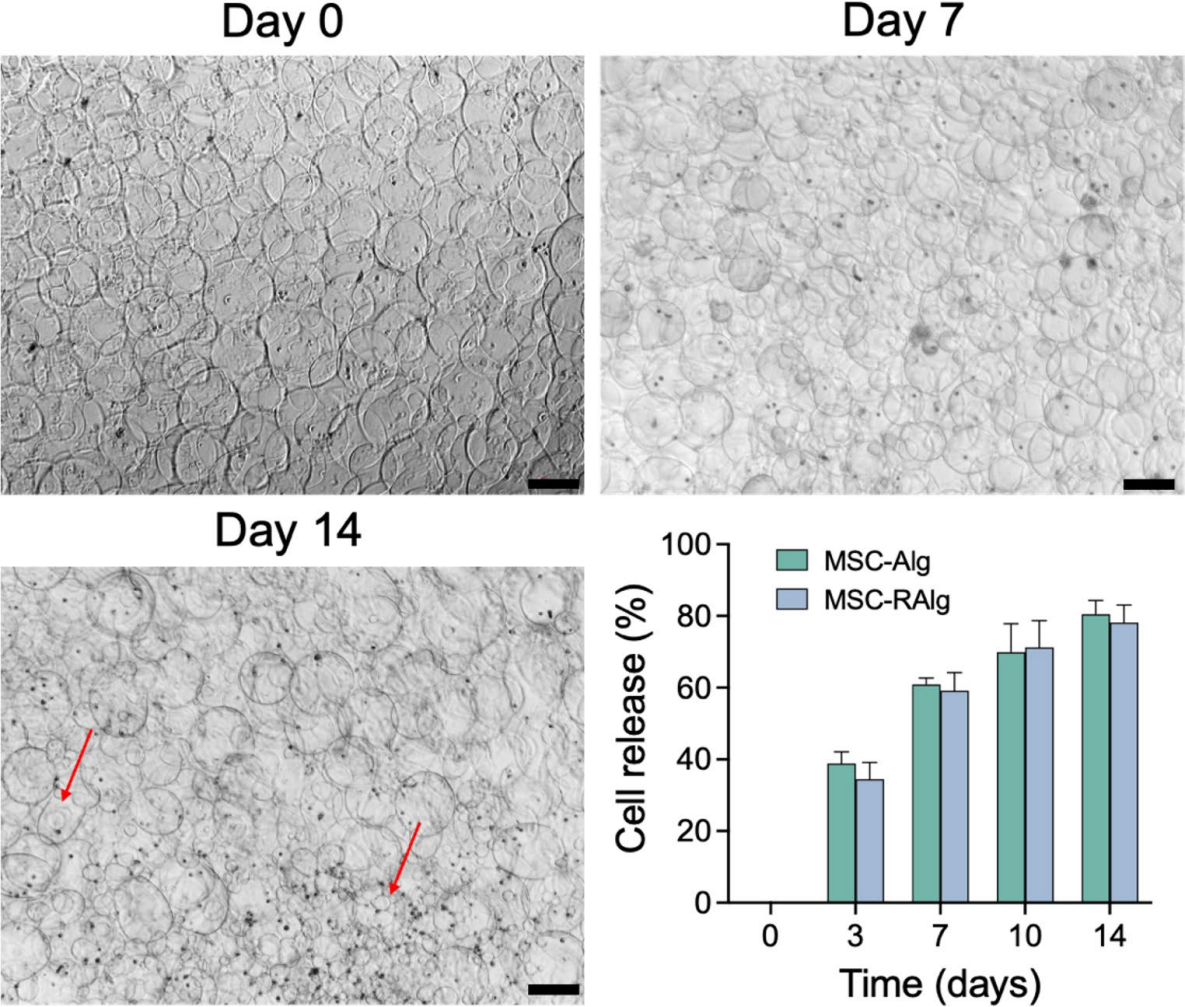
Cell release from MSC-loaded Alg microcapsules over 14 days. MSCs were encapsulated in Alg and RAlg microcapsules via electrospraying and incubated in phosphate-adjusted, serum free culture media with physiologic phosphate levels for 14 days. Released cells were quantified by manual counting under light microscopy. At day 0 (top left), most microcapsules remained intact and encapsulated. By day 7 (top right), partial degradation of microcapsules was observed, releasing approximately 59–61% of the cells. At day 14 (bottom left), most microcapsules degraded and shrunk, releasing 78–81% of the cells. No significant differences in cell release were observed between Alg and RAlg microcapsules (bottom right). Scale bar = 200 μm

The ability of the microcapsules to sustain MSC release was evaluated over a 14-day period. Both Alg and RAlg microcapsules exhibited comparable MSC release profiles throughout the study (Fig. 6). Microscopic imaging revealed gradual dissolution, degradation, and shrinkage of the microcapsules over time, consistent with controlled, time-dependent MSC release. Microcapsules remained largely intact at day 1, showed partial degradation with 59–61% release by day 7, and underwent extensive degradation with 78–81% release by day 14. No significant differences in degradation behavior or MSC release kinetics were observed between Alg and RAlg microcapsules.

## Discussion

The application of cell therapy in regenerative medicine remains limited by the lack of an optimized delivery system. In this study, we investigated cell microencapsulation using Alg-based microcapsules fabricated via electrospraying to develop an injectable delivery platform. We demonstrated that electrospraying is a versatile technique that enables precise control over microcapsule size of hydrogel formation to microcapsules. Furthermore, RAlg microcapsules possess ideal qualities well suited for the sustained delivery of MSCs while preserving cell viability.

Injectability of hydrogel delivery systems is fundamentally limited by microcapsule size, requiring precise control while preserving effective payload capacity. In this study, electrospraying enabled tunable and reproducible size modulation of microcapsules through systematic optimization of processing parameters, with applied voltage emerging as the dominant factor. Increasing voltage shifted the system from dripping to a stable cone–jet position, thereby enhancing electrostatic repulsion relative to surface tension yielding smaller droplets and microcapsules (Ahenkorah et al. 2024; Yao et al. 2012). Needle inner diameter further influenced microcapsule dimensions by defining the initial jet geometry which restricts the droplet size (Partovinia and Vatankhah 2019). Solution concentration modulated both microcapsule size and uniformity by affecting viscosity, jet deformability, and electrical conductivity. Lowering the concentration decreased microcapsule size with a deformed morphology because of surface instability and excessive droplet extension (Xu et al. 2006). Similarly, flow rate primarily controlled size dispersity by regulating solution residence time and charge exposure at the needle, destabilizing the electrical field and droplet atomization (Aminoroaya et al. 2024). Furthermore, our findings highlight the scalability of electrospraying RAlg in producing microcapsules compared to other fabrication methods where future application may aid in the delivery of cells, drugs, and other bioactive substances (Lai et al. 2024; McCrea et al. 2018; Wang et al. 2019). Compared to direct injection, RAlg microcapsules promote in vivo retention within target tissue and maintain free nutrients and therapeutic protein diffusion (Dhamecha et al. 2019). Together, these findings identify constrained yet reproducible parameters for electrospraying to produce injectable, size-controlled hydrogel microcapsules.

The physicochemical characteristics of Alg dictate how effective it is as a delivery system. Our findings revealed that Alg microcapsules have a rough, microporous surface architecture, a feature that facilitates the bidirectional flow and controlled release of nutrients and metabolites (Shamszadeh et al. 2022). Remnants of this porous network further indicated a highly ordered Alg polymer configuration, enabling the microcapsules to function as a scaffold while mimicking the native environment that supports cellular viability (Barcena et al. 2024; Sapkota et al. 2025). Both Alg and RAlg microcapsules exhibited high swelling stability which is mainly influenced by the concentration of calcium within the gel and affects molecular porosity (Savić Gajić et al. 2023). Furthermore, increasing the initial concentration led to a corresponding rise in viscosity, with pronounced differences between Alg and RAlg observed only at higher concentrations. The addition of a peptide may have increased the viscoelastic properties of Alg at higher concentrations related to the formation of attractive inter-particle clusters (Goldberg et al. 2021). The viscoelastic properties of hydrogel networks influence mechanotransduction and migratory cues of MSCs (Cameron et al. 2011; Chaudhuri et al. 2016). Viscoelasticity is crucial in future research to validate shear-thinning injectability, structural recovery after administration, and degradation rate (Guvendiren et al. 2011). Overall, our results are in accordance with an earlier study wherein, it was reported that RGD functionalization did not significantly affect the gelation, rheological properties, and injectability of Alg hydrogels (Mauri et al. 2018). Besides swelling and viscosity, other biomechanical properties such as compressive modulus and elastic modulus are important to evaluate as this may affect MSC mechanotransduction, fate, and survival (Huebsch et al. 2010). Polymer functionalization may influence these biomechanical properties by disrupting the homopolymeric blocks and alter mechanical stress response compared to unmodified alginate (Dalheim et al. 2019). Notably, the peptide-functionalized alginate did not compromise the encapsulation efficiency, indicating that RGD modification alters rheological behavior without disrupting the fundamental physical performance of the hydrogel delivery system.

MSCs were successfully encapsulated within Alg-based microcapsules, indicating the suitability of the electrospraying platform for efficient cell delivery. This format highlights the versatility of the system by enabling precise control over microcapsule formation and injectability, advantages not achievable when cells are delivered unencapsulated in a vehicle medium alone (Bolinas et al. 2025). Encapsulated cells predominantly exhibited a spherical morphology, consistent with physical confinement within a soft, non-adhesive hydrogel matrix (Hassan et al. 2013; Seifabadi et al. 2021). While this morphological change is regarded as a cell death in 2D culture, our findings showed that the cells remained viable similar with other Alg encapsulation studies (de Souza et al. 2021; Saberianpour et al. 2020). Importantly, this change does not limit the overall biocompatibility of Alg microcapsules but rather reflect the fundamental difference between 2D and 3D culture conditions. Notably, the RAlg microcapsules consistently preserved MSC viability more effectively than unmodified Alg. This enhanced survival is likely due to the presence of the RGD peptides, which promote integrin-mediated cell–biomaterial interactions and support pro-survival signaling pathways within the encapsulated microenvironment (Grigore et al. 2014; Lu et al. 2024; Shu et al. 2004). In general, MSCs are known as anchorage-dependent cells where in the absence of integrin-binding ligands they can undergo anoikis, an apoptotic process that results from the lack of cell and matrix interactions (Xu et al. 2026). MSCs interact with RGD via the α_v_β_3_ integrin, and cellular attachment leads to tyrosine phosphorylation and subsequent activation of focal adhesion kinase (FAK). FAK transmits adhesion dependent cell-survival signal pathways such as phosphatidylinositol 3-kinase (PI3K)/Protein kinase B (Akt or PKB) and mitogen-activated protein kinase (MAPK)/extracellular signal-regulated kinase (ERK) (Katoh 2024; Kudva et al. 2018; Salinas and Anseth 2008). Similar observations have been reported, where RGD functionalization improves cell viability within Alg matrices (Jeon and Alsberg 2013; Stahl et al. 2026). This highlights the potential of leveraging the advantages of electrospraying using RAlg for future development of cell encapsulation strategies.

To function effectively as a delivery system, microcapsules must regulate the release of encapsulated cells to sustain therapeutic effects. Our results indicate that the Alg-based microcapsules controlled the cell release through a mechanism mainly governed by intrinsic physicochemical characteristics of the Alg network, independent of RGD functionalization. Alg form hydrogels through ionic cross-linking of Alg chains by calcium ions. Under physiological conditions, the release of calcium due to exchange reactions with monovalent ions (sodium) or due to chelation of compounds (phosphate, citrate, lactate) results in gradual network destabilization externally. The gradual loss of calcium and matrix loosening releases the entrapped cells and permits encapsulated cells to migrate out (Boontheekul et al. 2005; K. Y. Lee and Mooney 2012). This mechanism is supported by the observed continuous decrease in microcapsule size over time, consistent with surface-mediated erosion rather than internal network collapse and abrupt bulk rupture. Importantly, while RGD conjugation significantly improved stem cell viability, our findings showed that it did not substantially alter the stability and degradation behavior of the Alg matrix. This allowed biological performance to be improved without compromising release kinetics. These findings of sustained release and enhanced cell survival suggest that RAlg microcapsules are a promising platform for injectable and localized stem cell delivery.

This study demonstrated the versatility of electrospraying Alg for producing injectable, size-controlled microcapsules with diverse applications. The flexibility of electrospraying parameters enabled precise tuning of microcapsule size. However, several limitations should be acknowledged. Only four parameters were evaluated, and air- or pressure-related factors that may disturb the jet trajectory and path length were not included. While rheological and viscometric parameters of the microcapsules were evaluated, comprehensive biomechanical characterization, including compression and elasticity testing, are necessary to further elucidate the impact of RGD functionalization on microcapsule physicochemical behavior. In addition, the study highlighted the biocompatibility of RAlg microcapsules in supporting cell viability and sustained release. However, the underlying mechanisms by which RAlg microcapsules promote cell survival were not investigated, and cell viability was assessed only for up to two weeks. Future studies should therefore focus to experimentally validate the mechanisms related to survival pathways, MSC adhesion, and signaling marker expression. Additionally, functional characterization of encapsulated MSCs was not performed and warrants further investigation, particularly with respect to immunomodulatory activity (e.g., cytokine secretion profiles), multilineage differentiation potential, stemness, and senescence markers. Furthermore, release behavior was evaluated in a quiescent in vitro system that does not replicate flow conditions or other dynamic factors present in vivo. Accordingly, future quantitative studies on dynamic systems to assess calcium ion release kinetics, hydrogel degradation rate, and cell migration should be explored. Despite these limitations, our findings provide valuable insights into tailoring delivery systems using electrosprayed microcapsules and underscore the need to explore alternative biomaterial blends suited to specific applications. Overall, this work contributes to establishing a framework for optimizing microcapsule fabrication and for developing Alg-based hydrogel delivery systems platforms with translational potential.

## Conclusion

Bridging the translational gap between preclinical studies and clinical trials remains a pressing problem to current progress in MSC-based cell therapy. Injectable cell delivery systems provide a promising solution to overcome these limitations. In this study, we present an electrospraying-based approach for microencapsulating MSCs within RAlg microcapsules. Precise optimization of electrospraying parameters enabled the production of highly uniform microcapsules that improved cell viability and supported sustained in vitro release. This delivery platform provides robust mechanical protection, enhances cell survival, and prolongs cell retention, while remaining scalable, reproducible, and clinically compatible. Ultimately, this study this study shows the applicability of electrospray technique to develop a cell-based delivery system which offers a promising and translatable solution for advancing stem cell–based regenerative medicine.

## Data Availability

The data used to support the findings of this study are available from the corresponding author upon reasonable request.
